# Protein Self-Assembly States Modulate Lithium Carbonate Biomineralization: From Ion Chelation to Nucleation Sites

**DOI:** 10.1021/acs.biomac.5c01601

**Published:** 2025-12-12

**Authors:** Zhichun Lin, Yizhen Yan, Archie Hunter, Huaiyu Yang

**Affiliations:** Department of Chemical Engineering, 5156 Loughborough University, Loughborough, Leicestershire LE11 3TU, United Kingdom

## Abstract

Understanding protein–salt interactions is important for controlling crystallization, including biomineralization, biopharmaceutical purification, biocatalytic enzymes, and environmental biointerfaces. This study for the first time investigated interactions between three proteins (lysozyme, red fluorescence protein, and bovine hemoglobin) and Li_2_CO_3_ (formation from the reaction of LiCl with Na_2_CO_3_) under different protein and salt concentrations. For the crystallization of Li_2_CO_3_, at low supersaturation (*S*), the proteins inhibited Li_2_CO_3_ nucleation by 20–40% through chelation. At high *S*, the proteins accelerated nucleation by 10–40%. The dual effects of the protein on Li_2_CO_3_ biomineralization have been discussed. With the increase *S* of Li_2_CO_3_, the dispersion state of proteins in solution undergoes a transition from dimers to oligomers and finally to aggregates. In all ranges of *S*, the protein reduced the agglomeration of Li_2_CO_3_ crystals. In lysozyme crystallization, increasing the Li_2_CO_3_ concentration yielded a larger number of smaller crystals. At equal concentration of lysozyme, twice more of LiCl and Na_2_CO_3_ in the solution led to more than 5 times the crystal number and 5 times smaller average crystal size. The interactions among protein molecules, salt ions in solution, and Li_2_CO_3_ crystals have been discussed. Dynamic light scattering measurements and the fluorescence microscopy image suggest that the dual effect of proteins on Li_2_CO_3_ crystallization at different supersaturation levels is associated with protein molecular aggregation under varying salt concentrations, resulting in both thermodynamic and kinetic influences on the crystallization process.

## Graphical abstract

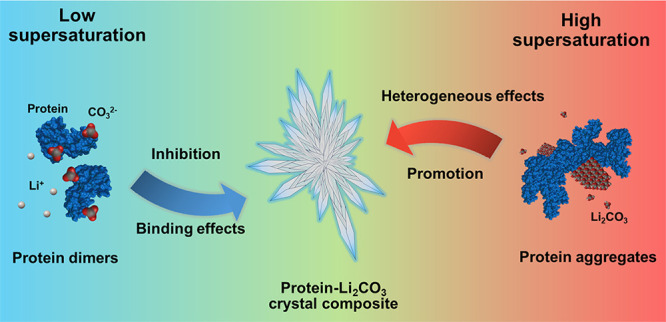

## 1 Introduction

Crystallization is a key process in chemical and material engineering, widely used in the recovery and purification of pharmaceuticals, organic compounds, and inorganic salts.^1^ The crystallization thermodynamics and kinetics are influenced by various parameters like temperature, pH, solvent properties, and externally introduced additives.^2,3^ Inorganic salts play a dual role in crystallization processes involving biological molecules. In protein crystallization, molecular-level interactions, such as protein–ion interactions, can influence nucleation rates, crystal habits, and polymorphs of protein crystals.^4–7^ Inorganic salts, such as NaCl, CaCl_2_, and (NH_4_)_2_SO_4_, can result in the decrease of the protein solubility, which have been widely employed as precipitants in protein crystallization.^8–10^ The selection of inorganic salt ions is often guided by the Hofmeister series, which ranks ions based on their effects on protein solubility,^11^ in the order of CO_3_^2–^> PO_4_^3–^> SO_4_^2–^> C_2_H_3_O_2_^–^> Cl^–^> Br^–^> ClO_4_^–^> I^–^> SCN^–^ for anions and in the order of NH_4_^+^ > Rb^+^ > K^+^ > Na^+^ > Li^+^ > Ca^2+^ > Mg^2+^ > Zn^2+^ > Ba^2+^ for cations. Chaotropic ions, such as SCN^–^ and I^–^, destabilize proteins by disrupting hydration layers and weakening hydrophobic interactions, while structure-making ions, such as SO_4_^2–^ and HPO_4_^2–^, significantly lower solubility by enhancing water molecule ordering and promoting competitive dehydration effects.^12^ On the other hand, biological macromolecules exhibit potential in modulating the nucleation and growth dynamics of inorganic crystals, causing biomineralization.^13^ For example, proteins such as bovine serum albumin can influence the nucleation of NaCl, KCl, and MgCl_2_ crystals and regulate crystal morphology depending on protein concentrations.^14^ Protein–salt interactions can also control the morphology of inorganic crystals through electrostatic effects and structural matching.^15^ Negatively charged protein surfaces, for instance, attract Ca^2+^ ions, inhibiting calcite face growth while promoting the formation of calcium carbonate vaterite.^16^ The structural matching mechanism relies on spatial complementarity between specific protein surface residues and the inorganic lattice. Aspartic acid (Asp) residues, through their carboxyl groups, stabilize prenucleation clusters, delay nucleation, and alter crystal morphology,^17^ while ferritin uses glutamic acid residues at its active sites to promote iron mineralization and microcrystal formation.^18^

Lithium carbonate (Li_2_CO_3_), a key precursor for battery electrode fabrication and ceramic/glass manufacturing,^19^ is conventionally synthesized through precipitation via reagent addition (sodium carbonate) or gas–liquid reaction (carbon dioxide bubbling) in lithium-rich solutions.^20^ The precipitation crystallization of Li_2_CO_3_ is driven by the following reaction:

$$2\mathrm{LiCl}+{{\mathrm{CO}}_{3}}^{2-}\to {\mathrm{Li}}_{2}{\mathrm{CO}}_{3}↓+2{\mathrm{Cl}}^{-}$$ 1

Optimizing the crystallization of Li_2_CO_3_ is essential for cost-effective lithium recovery from brine resources. In addition to the critical parameters of temperature, concentration, and pH, the role of various additives in modulating the crystallization of Li_2_CO_3_ has been examined in recent studies.^21^ Sodium hexametaphosphate (SHMP), sodium tripolyphosphate (STPP), and polymaleic acid (PMA) have been reported to induce microcrystalline, spherical Li_2_CO_3_ structures.^22^ Polyelectrolytes and surfactants, such as poly­(acrylic acid) (PAA), sodium dodecyl sulfate (SDS), polyethylenimine (PEI), and polyethene glycol (PEG), can modulate Li_2_CO_3_ crystal size and reduce agglomeration.^23,24^ Crystal agglomeration is one of the key challenges in industry, which directly impacts product purity, particle size distribution, and flow properties.^25^ It is reported that protein–salt interactions were able to control the morphology and agglomeration of salt crystals.^26–29^ However, the underlying mechanisms for many of these complex crystallization processes remain poorly understood, and no investigations on the interactions between proteins and Li_2_CO_3_ or their effects on its biomineralization behavior have been reported.

This study investigates the crystallization of Li_2_CO_3_ with reactant concentrations of 0.3–2 M LiCl and 0.2–1 M Na_2_CO_3_. Lysozyme of 1–50 mg/mL, monomeric red fluorescence protein (mRFP), and bovine hemoglobin were applied to investigate interactions between protein and Li_2_CO_3_ under mildly alkaline and acidic conditions. Lysozyme is a widely used model protein in biomineralization and crystallization. Bovine hemoglobin and mRFP have different molecular sizes and isoelectric points (pI) compared to lysozyme, providing a more comprehensive system for investigation. The shapes of crystals of Li_2_CO_3_, lysozyme, and the mixture were observed. Crystal numbers, distributions, and time of nucleation were measured and analyzed. Distributions of protein and inorganic salts were observed via fluorescence microscopy. Particle size changes in the initial stage of crystallization were observed by DLS. The mechanisms of protein adsorption on inorganic salts and interactions between proteins and inorganic salts were discussed.

## 2 Experimental Section

### 2.1 Materials

Ni-NTA agarose for mRFP purification was purchased from Thermo Scientific. Chicken egg white lysozyme (70,000 U·mg^–1^), bovine hemoglobin (BHb), sodium carbonate (≥99.5%), lithium chloride (≥99.0%), anhydrous sodium acetate (≥99.0%), and acetic acid (≥99.5%) were purchased from Sigma-Merck. Calcium chloride dihydrate (assayed 100.4%) and magnesium chloride hexahydrate (assayed 99.4%) were obtained from VWR International. Red fluorescent protein (mRFP) was expressed in and purified from *Escherichia coli* BL21 transformed with the mRFP plasmid. Ultra-high-purity deionized water (18.2 MΩ·cm^–1^) was obtained from the Milli-Q Water system (Millipore Corporation). No further purification steps were required for all agents.

### 2.2 Methods

Plasmid of mRFP was transferred to *E. coli* BL21. Cells were grown in LB broth at 37 °C for 2 h, and mRFP protein was expressed at 25 °C for 20 h. The protein was purified through a Ni-NTA column and ultrafiltration. The concentration of mRFP was determined through a Thermo Scientific NanoDrop UV spectrophotometer. In experiments conducted in a neutral environment, proteins (lysozyme, bovine hemoglobin, and mRFP) were dissolved in water. Na_2_CO_3_ solutions were prepared at concentrations of 0.2, 0.4, 0.6, 0.8, and 1 M, and LiCl solutions were prepared at 0.4, 0.6, 0.8, 1, and 1.2 M. In experiments in acid solution, lysozyme (10, 25, 50 mg/mL) and salts (0.5, 0.75, 1 M Na_2_CO_3_ and 1, 1.5, 2 M LiCl) were dissolved in 0.1 M C_2_H_4_O_2_–C_2_H_3_NaO_2_ aqueous buffer. The pH was adjusted to 4.3 with a pH meter (a SciQuip 920 precision). All buffers and solutions were filtered by using a 0.2 μm cellulose acetate filter. The supersaturation of Li_2_CO_3_ crystallization with or without lysozyme was in the range of 1.2–6.0. Based on the influences of lysozyme, the supersaturation of Li_2_CO_3_ crystallization with mRFP and hemoglobin in the solution was designed as 2.4 and 4.8.

Hanging drop crystallization experiments were performed in 24-well plates. In these experiments, LiCl at a concentration of 1.2 M was used in the reservoir solution.^29^ Equal volumes of Na_2_CO_3_, LiCl, and protein were uniformly mixed by adding 0.5 μL of each solution onto a cover slide. The droplets and the reservoir solution were sealed in a tube with silicone grease. Due to concentration gradients, vapor continuously diffused from the droplets into the reservoir solution, gradually increasing the solute concentration in the droplets. Each condition was repeated with 16 droplets and the plates with droplets were maintained at 20 °C in a thermostatically controlled chamber (Loviband). Crystallization was monitored using a phase contrast microscope (UltraBIO-6PH), with an observation interval of every 10 min during the first 30 min and then every 30 min until 5 h. After that, observations were continued every 1 h until the first visible crystal appeared.

The fluorescence images of Li_2_CO_3_ with proteins were captured under a fluorescence microscope (Nikon Eclipse TE-300). SEM images were captured through a JSM-7100F after coating the samples with Pd powders in a turbomolecular pumped combined sputter coater (Q150T ES). Dynamic light scattering (DLS) analysis was carried out with a Malvern Zetasizer Ultra. DTS0012 disposable cuvettes were applied to hold 1.2 mL of the sample.^30^ Each DLS measurement continued for 30 s at 20 °C and was conducted 5 times to obtain an average. Back scattering mode was applied, and data were modeled using a multiple-peaks model.

## 3 Results

Figure 1 shows that the Li_2_CO_3_ nucleation time decreases with increasing LiCl (0.3–0.6 M) or Na_2_CO_3_ (0.2–0.5 M) concentrations, with or without lysozyme in the solution at neutral pH. This trend was due to Reaction 1 shifting toward Li_2_CO_3_ precipitation. The influence of lysozyme was dependent on the concentration of the salts in the solution. The lysozyme in the solution delayed the nucleation occurrence in droplets of low salt concentrations, as shown in Figure 1, and distributions of nucleation times in the Supporting Information. The average nucleation time of Li_2_CO_3_ was 16.7 h with 1 mg/mL lysozyme and 11.8 h without lysozyme, in the droplets with conditions of 0.4 M Na_2_CO_3_ and 0.3 M LiCl. With an equal concentration of 0.4 M Na_2_CO_3_ and increase in the LiCl concentration, the difference in nucleation time with and without lysozyme became smaller. At 0.3 M LiCl, the nucleation time of the droplet with lysozyme increased by about 5 h, while at 0.4 M LiCl, the nucleation time increased by about 3 h.

**Figure 1:**
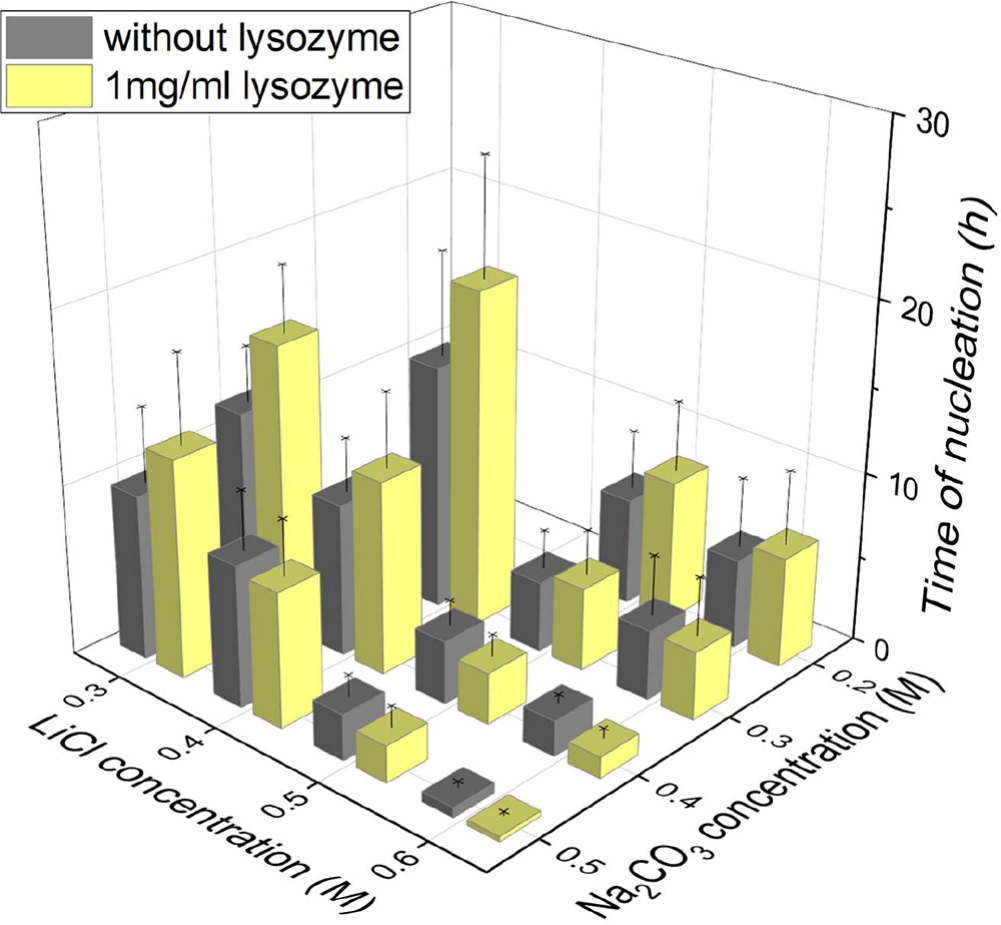
Nucleation times of Li_2_CO_3_ in the droplets of 0.3–0.6 M LiCl and 0.2–0.5 M Na_2_CO_3_, with the addition of 1 mg/mL lysozyme and without lysozyme.

At high concentrations of salts, the lysozyme accelerated nucleation. In droplets of 0.5 M LiCl, the nucleation time of Li_2_CO_3_ in the droplets with lysozyme was about 0.6 h shorter than those without lysozyme. The effect of accelerating nucleation by lysozyme became stronger with a further increase in the salt concentrations. In the droplets of 0.6 M LiCl, the nucleation time of Li_2_CO_3_ in the droplets with lysozyme was about 0.8 h shorter than those without lysozyme. The nonmonotonic relationship of nucleation with lysozyme in the salt of different concentrations was also observed under fixed concentrations of LiCl and increasing concentrations of Na_2_CO_3_. At equal 0.5 M LiCl with low Na_2_CO_3_ concentrations, the droplets tended to nucleate slower with lysozyme (2 h delay at 0.2 M Na_2_CO_3_) than those without lysozyme. At high concentrations of Na_2_CO_3_, the droplets tend to nucleate faster (0.6 h earlier at 0.5 M Na_2_CO_3_) with lysozyme than those without.

Figure 2A–F shows the images of Li_2_CO_3_ crystals observed after 144 h at low, middle, and high salt concentrations with and without protein, and the conditions of the images are marked in Figure 2G. Figure 2A shows that when salt concentrations were low, under conditions of 0.4 M LiCl and 0.3 M Na_2_CO_3_, the Li_2_CO_3_ crystals tended to form lamellar crystals, and most of them agglomerated. At this salt condition, with 1 mg/mL lysozyme, most of the Li_2_CO_3_ formed as single lamellar crystals, as shown in Figure 2D. Figure 2B shows that when the salt concentration reached a moderate level, under conditions of 0.6 M LiCl and 0.3 M Na_2_CO_3_, the stick-shaped Li_2_CO_3_ crystals formed agglomerations. At this salt condition, with 1 mg/mL lysozyme, most of the Li_2_CO_3_ formed needle-like crystals, shown in Figure 2E, exhibiting a lower degree of agglomeration.

**Figure 2:**
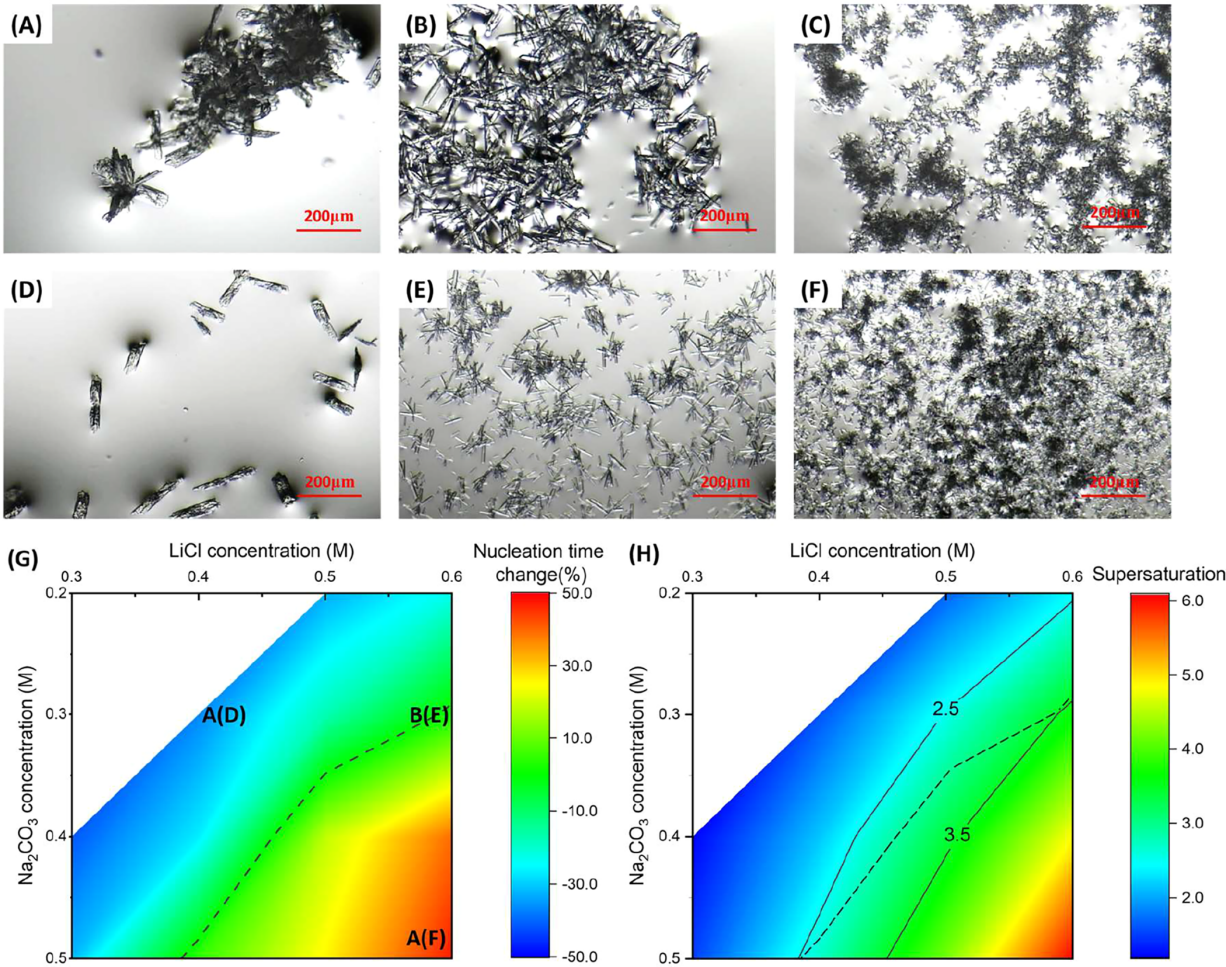
Optical microscopy of Li_2_CO_3_ crystals after 144 h in droplets of 0.4 M LiCl and 0.3 M Na_2_CO_3_ without lysozyme (A) and with 1 mg/mL lysozyme (D), 0.6 M LiCl and 0.3 M Na_2_CO_3_ without lysozyme (B) and with 1 mg/mL lysozyme (E), 0.6 M LiCl and 0.5 M Na_2_CO_3_ without lysozyme (C), and with 1 mg/mL lysozyme (F). (G) Percentage nucleation times change of Li_2_CO_3_ in the droplet with lysozyme compared to the droplets without lysozyme, at equal salt concentrations. (H) Supersaturation of Li_2_CO_3_ under conditions of various salt concentrations. The dashed lines are guidance lines for similar nucleation time conditions.

Figure 2C shows that in droplets with high salt concentrations, under conditions of 0.6 M LiCl and 0.5 M Na_2_CO_3_, some needle-shaped crystals were obtained, with obvious agglomerations. At this salt condition, with 1 mg/mL lysozyme, the size and shape of Li_2_CO_3_ crystals were similar to those obtained without lysozyme. There were lighter agglomerations of Li_2_CO_3_ crystals, with more separated individual Li_2_CO_3_ crystals, obtained with lysozyme (Figure 2F) compared to those Li_2_CO_3_ crystals obtained without lysozyme (Figure 2G).

Figure 2G shows the comparison of nucleation times with and without lysozyme at equal concentrations of the salts. At lower salt concentrations (LiCl ≤ 0.4 M, Na_2_CO_3_ ≤ 0.4 M), nucleation times in the droplets with lysozyme were about 20–40% longer than those without lysozyme, respectively, showing an inhibiting effect. At higher concentrations (LiCl ≥ 0.5 M, Na_2_CO_3_ ≥ 0.4 M), the nucleation times in the droplets with lysozyme were about 10–40% shorter than those without lysozyme, respectively, showing an accelerating effect. The transition region, where the inhibiting effect of lysozyme on nucleation of Li_2_CO_3_ shifted to an accelerating effect, was observed between the conditions of 0.4 M LiCl, 0.5 M Na_2_CO_3_ and the conditions of 0.6 M LiCl, 0.3 M Na_2_CO_3_, as green areas in Figure 2G with a dashed curve for guidance of the middle of the region. The overall trend of nucleation time changes is similar to the patterns of supersaturation, as shown in Figure 2H. The supersaturation ratio *S* of Li_2_CO_3_ is defined as

$$S=\frac{(C_{{\mathrm{Li}}^{+}}{)}^{2}C_{{{\mathrm{CO}}_{3}}^{2-}}}{\mathrm{Ksp}}$$ 2

which is calculated based on the solubility of Li_2_CO_3_, where Ksp = 0.0297 for Li_2_CO_3_ at 20 °C.^31^ Under all experimental concentrations, all solutions in droplets were supersaturated (*S* > 1). The transition region corresponding to Figure 2H was presented as the supersaturation region between 2.5 and 3.5 in Figure 2G. The guidance dashed line in Figure 2H corresponds to the dashed line in Figure 2G. When the supersaturation of Na_2_CO_3_ and LiCl crystallization solution was low (<2.5), nucleation was inhibited by lysozyme. When supersaturation was high (>3.5), nucleation was accelerated with the addition of lysozyme.

Figure 3 shows the DLS results in the initial stage (within 20 min) after mixing of LiCl and Na_2_CO_3_ in the solutions with 1 mg/mL lysozyme. In the solutions with 0.4 M LiCl and 0.2 M Na_2_CO_3_, 0.6 M LiCl and 0.3 M Na_2_CO_3_, and 0.8 M LiCl and 0.4 M Na_2_CO_3_, there are three peaks that are consistent with the solution with the reported distribution of dissolved lysozyme, without salt/protein precipitation. The first peak, observed at about 4–5 nm, corresponds to the lysozyme dimer, which is twice the size of the monomer.^32–34^ The second peak, around 30–40 nm, indicates the presence of particles comprising several lysozyme molecules, which are lysozyme oligomers. The third peak, appearing above 100 nm, reflects the formation of aggregates of many lysozyme molecules in the solution. The size distributions are based on the intensity; therefore, the number of lysozyme oligomers was low, and the number of aggregates was very low.

**Figure 3:**
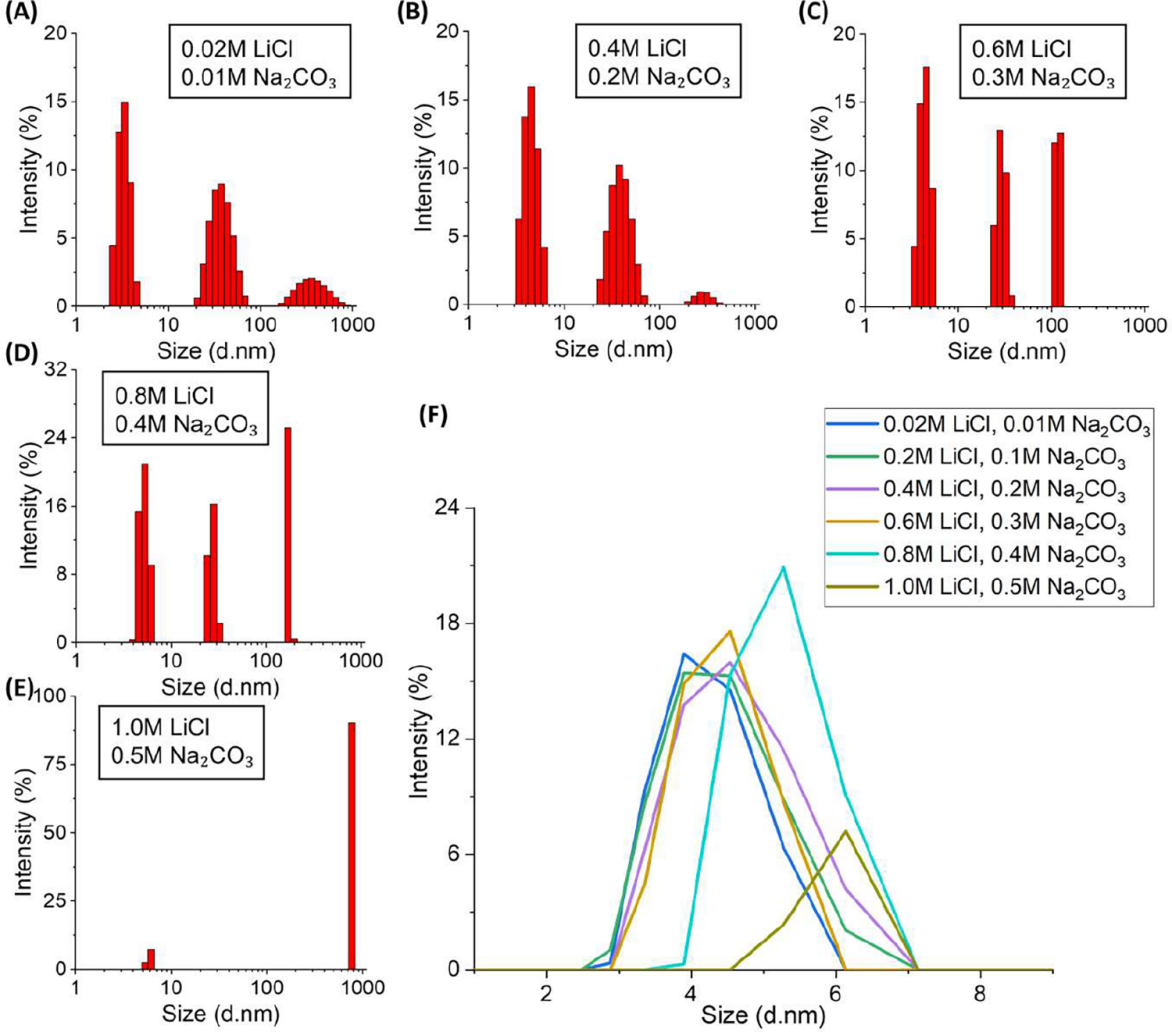
Intensity-based particle distributions of lysozyme molecules and their aggregations by DLS in the solutions of 1 mg/mL lysozyme with (A) 0.02 M LiCl and 0.01 M Na_2_CO_3_, (B) 0.4 M LiCl and 0.2 M Na_2_CO_3_, (C) 0.6 M LiCl and 0.3 M Na_2_CO_3_, (D) 0.8 M LiCl and 0.4 M Na_2_CO_3_, (E) 1 M LiCl and 0.5 M Na_2_CO_3_, and (F) comparison of these conditions in the range of 1–10 nm.

With the increase of the concentration in the solutions from 0.02 M LiCl and 0.01 M Na_2_CO_3_ (Figure 3A) to 0.4 M LiCl and 0.2 M Na_2_CO_3_ (Figure 3B), there is no obvious change in the lysozyme dimer. When the concentration increases to 0.6 M LiCl and 0.3 M Na_2_CO_3_ (Figure 3C), all peaks of lysozyme dimer size become much narrower, with a significant increase in the intensity of aggregates above 100 nm. There is an obvious change in the solution at 0.8 M LiCl and 0.4 M Na_2_CO_3_ (Figure 3D), with much less intensity in the peaks for the lysozyme dimer and oligomers and larger intensity in the peaks for the aggregates in solution of 1.0 M LiCl and 0.5 M Na_2_CO_3_ (Figure 3E), a new dominant peak appears at about 750 nm; the peak for the lysozyme dimer obviously decreases, and the peaks for the lysozyme oligomers and lysozyme aggregates both disappear. The presence of the lysozyme dimer peak was likely attributable to the influence of carbonate ions, as carbonate radicals could promote biomolecular cross-linking.^35^

Figure 3F shows that the size distributions of the lysozyme dimer in the solution from 0.02 M LiCl and 0.01 M Na_2_CO_3_ to 0.6 M LiCl and 0.3 M Na_2_CO_3_ are very similar to each other, indicating limited or no change of the lysozyme dimer. The changes were observed in the solutions 0.8 M LiCl and 0.4 M Na_2_CO_3_, and 1.0 M LiCl and 0.5 M Na_2_CO_3_, which was consistent with Figure 3D,E, indicating that the change of the lysozyme state in the solution starts between 0.6 M LiCl and 0.3 M Na_2_CO_3_ and between 0.8 M LiCl and 0.4 M Na_2_CO_3_. These salt conditions were in a similar range as the transition region (Figure 2G) of the nonmonotonic relationship between lysozyme and the salt nucleation.

The change in the result between the conditions involving 0.8 M LiCl and 0.4 M Na_2_CO_3_ and involving 1.0 M LiCl and 0.5 M Na_2_CO_3_ was consistent with the obvious change in the crystallization process. In the solution with 0.8 M LiCl and 0.4 M Na_2_CO_3_ and lower salt concentrations, the lysozyme remained relatively stable. There was no precipitation observed over 8 h, consistent with the behavior of lysozyme colloidal clusters.^36^ In a solution of 1.0 M LiCl and 0.5 M Na_2_CO_3_ with lysozyme, there was a large quantity of precipitates within 20 min, as the droplet solution became turbid. The phenomena were consistent with the DLS measurement in Figure 3E, where much larger aggregates formed in a short time, approaching a monodisperse state. These precipitates in the solution appeared flocculent under the microscope, mainly resulting from the salted out of the lysozyme (instead of Li_2_CO_3_ precipitation).^37,38^ This is because there was no peak in the range of 10–1000 nm in the solutions of 1.0 M LiCl and 0.5 M Na_2_CO_3_, without lysozyme, by the DLS measurements during several hours.

Without lysozyme, Li_2_CO_3_ crystals formed spherical agglomerations, and each agglomeration included 10 to 30 lamellar crystals (Figure 4A). As lysozyme was unstable and easy to denature even at very low concentrations under neutral pH conditions without a buffer solution, the solution with acetic acid and sodium acetate as buffer was adjusted to an acidic environment to further investigate the influence of higher concentrations of lysozyme on the crystallization of Li_2_CO_3_. As in the solution with acidic conditions, the solubility of Li_2_CO_3_ increased. Therefore, the salt concentrations required for Li_2_CO_3_ to nucleate at the same period were much higher than those in the solution under neutral pH conditions. With a lower pH at 4.3, with 10 mg/mL lysozyme in the solution, fewer agglomerations appeared, and the Li_2_CO_3_ lamellar crystals stayed as single and separated crystals, as shown in Figure 4B.

**Figure 4:**
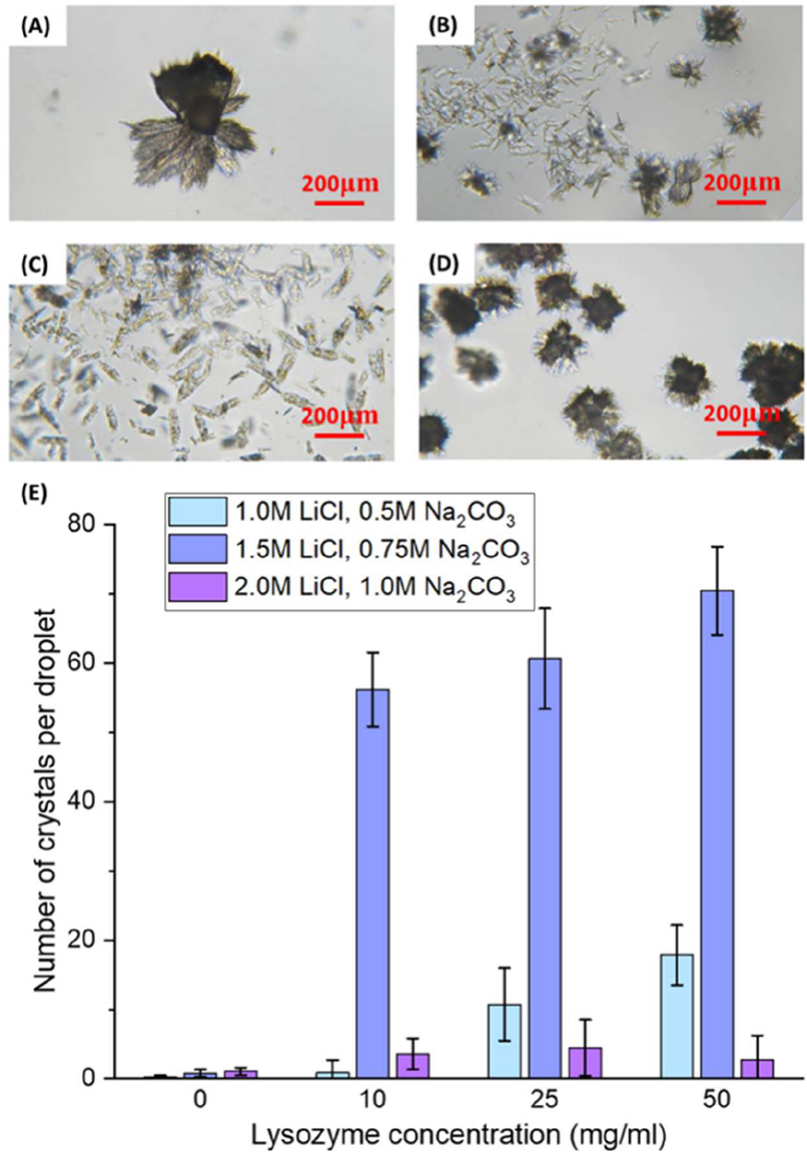
Spherulite and lamellar crystals of Li_2_CO_3_ obtained at pH = 4.2 at 5 h in 1.5 M LiCl and 0.75 M Na_2_CO_3_ without lysozyme (A) and with 10 mg/mL lysozyme (B), in 1.5 M LiCl and 0.75 M Na_2_CO_3_ with 50 mg/mL lysozyme (C), and in 2.0 M LiCl and 1.0 M Na_2_CO_3_ with 50 mg/mL lysozyme (D). (E) Average number of crystals at 5 h in droplets of 1.0, 1.5, and 2.0 M LiCl and 0.5, 0.75, and 1.0 M Na_2_CO_3_ (C_LiCl_: C_Na2CO3_ = 2:1) with 0–50 mg/mL lysozyme.

An increase in lysozyme concentration to 50 mg/mL resulted in larger sizes of dispersed Li_2_CO_3_ lamellar crystals (Figure 4C) due to the lysozyme with an antiagglomeration effect in the solution. With a further increase in the salt concentrations (2.0 M LiCl and 1.0 M Na_2_CO_3_), the antiagglomeration effect of the lysozyme was very limited, and the Li_2_CO_3_ crystals always formed spherical agglomerations, as shown in Figure 4D. This may be because, at high salt concentrations, the nucleation rate of Li_2_CO_3_ was very fast, leading to numerous Li_2_CO_3_ tiny crystals in the solution, and, however, the available lysozyme molecules seem to be insufficient to separate or prevent their agglomeration.

Figure 4E shows that with an increase in salt concentrations from 1.0 M LiCl and 0.5 M Na_2_CO_3_ to 1.5 M LiCl and 0.75 M Na_2_CO_3_, more crystals formed in the solution within 5 h due to high supersaturation. At fixed salt concentrations, increasing the lysozyme concentration from 10 to 50 mg/mL resulted in a significant increase in crystal number, which is evidenced by Figure 4A,B. This trend can be attributed to the more single crystals with less agglomeration at higher lysozyme levels, as observed in solutions with 1 M LiCl/0.5 M Na_2_CO_3_ and 1.5 M LiCl/0.75 M Na_2_CO_3_. In the droplets of 2.0 M LiCl and 1.0 M Na_2_CO_3_, with an increase in the lysozyme concentrations from 25 to 50 mg/mL, there was no increase in the number of crystals, as most crystals formed agglomerations quickly after mixing.

Figure 5A shows the positive and negative influences of different proteins on the nucleation of Li_2_CO_3_, dependent on the supersaturation. With the addition of 1 mg/mL mRFP in solution with 0.6 M LiCl and 0.2 M Na_2_CO_3_ under alkaline environments, where the supersaturation of Li_2_CO_3_ was relatively low, the nucleation time was longer than that in the solution without mRFP. This trend was consistent with the results observed for bovine hemoglobin and with the effect of lysozyme, both of which showed slower nucleation at Li_2_CO_3_ supersaturations of about 2.4. Under a high salt concentration of 0.6 M LiCl with 0.4 M Na_2_CO_3_ with supersaturation about 4.8, nucleation occurred after a shorter period in the droplets with 1 mg/mL mRFP and hemoglobin than in the droplets without mRFP and hemoglobin. This trend was consistent with the influence of lysozyme under high supersaturation of Li_2_CO_3_ as well. Figure 5B shows that, in the condition with 0.6 M LiCl and 0.4 M Na_2_CO_3_, the nucleation of Li_2_CO_3_ occurred faster with an increase in the concentration of mRFP in the droplet. The average nucleation times were about 2.0 h, 1.7 h, 1.1, and 0.4 h in the solution containing 0 (without protein), 1, 5, and 25 mg/mL mRFP in the solution, respectively.

**Figure 5:**
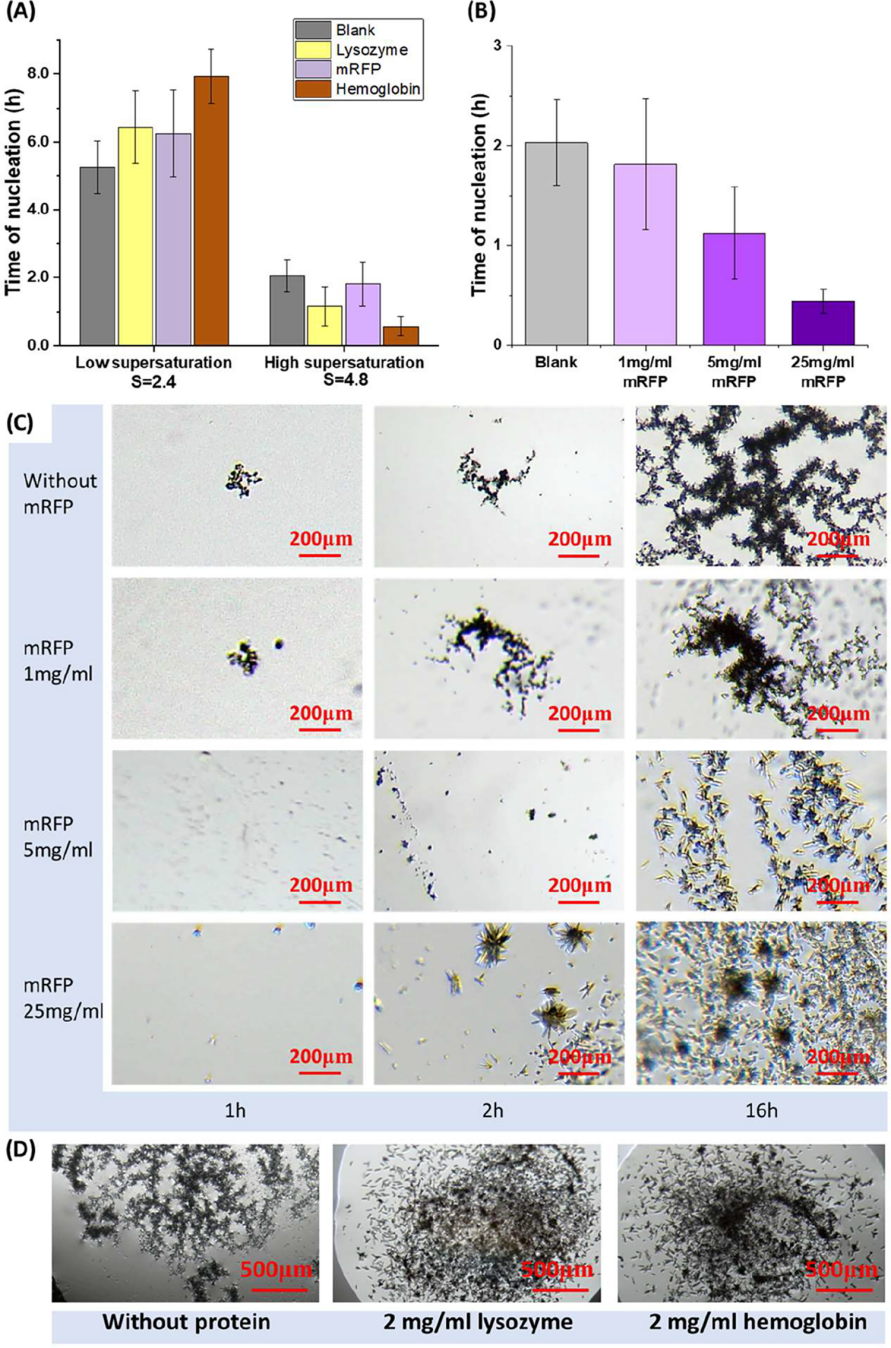
(A) Nucleation times of Li_2_CO_3_ in the droplets of 0.6 M LiCl and 0.2 M Na_2_CO_3_ at *S* = 2.4 with 1 mg/mL lysozyme, mRFP, and hemoglobin and in the droplets of 0.6 M LiCl and 0.4 M Na_2_CO_3_ at *S* = 4.8 with proteins. (B) Nucleation time in droplets of 0.6 M LiCl and 0.4 M Na_2_CO_3_, with 0, 1, 5, and 25 mg/mL mRFP. (C) Li_2_CO_3_ crystals in droplets of 0.6 M LiCl and 0.4 M Na_2_CO_3_ with 0, 1, 5, and 25 mg/mL mRFP at 1, 2, and 16 h. (D) Li_2_CO_3_ crystals in droplets of 0.6 M LiCl and 0.4 M Na_2_CO_3_ without protein, with 2 mg/mL lysozyme and 2 mg/mL hemoglobin, captured at 24 h.

Figure 5C shows the number of Li_2_CO_3_ crystals increased within 16 h in the solution of 0.6 M LiCl and 0.4 M with 0–25 mg/mL mRFP. Dendritic structures of Li_2_CO_3_ crystal were observed in the droplets with 1 mg/mL mRFP in the solution and without mRFP. The agglomeration of Li_2_CO_3_ crystals was markedly reduced at 16 h in the solution with 1 mg/mL mRFP than in the solution without mRFP; however, there were no obvious differences within the initial 2 h. With 5 mg/mL mRFP, the crystals became more uniformly distributed in the solution compared with those in the solution with 1 mg/mL mRFP in the solution and without mRFP. The individual crystals were larger, with much less dendritic agglomeration. At 25 mg/mL mRFP, some large spherulite crystals were observed within 2 h. After that, a large quantity of small needle crystals appeared within 16 h. The results were similar in lysozyme and bovine hemoglobin, where dendritic agglomeration was inhibited, as shown in Figure 5D.

With very low concentrations of LiCl and Na_2_CO_3_, there was no driving force for Li_2_CO_3_ to nucleate, and only lysozyme crystallized under the conditions in Figure 6A. Only a few lysozyme crystals formed under low protein or salt concentrations, as the supersaturation of lysozyme was low. In most droplets of 10 mg/mL lysozyme with 0.2 M LiCl and 0.1 M Na_2_CO_3_, there was only one large single crystal (about 500 μm) in each droplet. With an increase in salt or lysozyme concentrations, more small crystals (<100 μm, 5 times smaller) were observed in the droplet with 40 mg/mL lysozyme, 0.4 M LiCl, and 0.2 M Na_2_CO_3_. All of these crystals have similar shapes. In the droplets of 40 mg/mL lysozyme, with 0.2 M LiCl and 0.1 M Na_2_CO_3_, there were more crystals than in the droplets with a lower lysozyme concentration and the same salt concentration. Figure 6B shows that there were more than 10 times the number of crystals in the droplets of 40 mg/mL lysozyme, with 0.3 M LiCl and 0.15 M Na_2_CO_3_ and 0.4 M LiCl and 0.2 M Na_2_CO_3_ than in the droplets of 20 and 10 mg/mL lysozyme with the same salt concentrations, respectively. At 0.2 M LiCl and 0.1 M Na_2_CO_3_, there were only 10 crystals on average under 10 mg/mL lysozyme in the droplet. Under 20 and 40 mg/mL lysozyme, the average number of crystals increased to 66 and 184 (6 to 18 times), respectively. These results correspond to the effect of salts on lysozyme, such as NaCl and CaOx,^29,39^ as the increase in salt concentration of Li_2_CO_3_ leads to an increase in supersaturation of lysozyme.^29,40^ However, a small amount of protein precipitate was observed in some of the droplets after several days. According to the Hofmeister series, CO_3_^2–^ belongs to the strong salting-out ions and exhibits a high charge density. Although Li^+^ has a relatively weak salting-out ability, CO_3_^2–^ possesses a strong capability to sequester water molecules surrounding proteins.^41,42^

**Figure 6:**
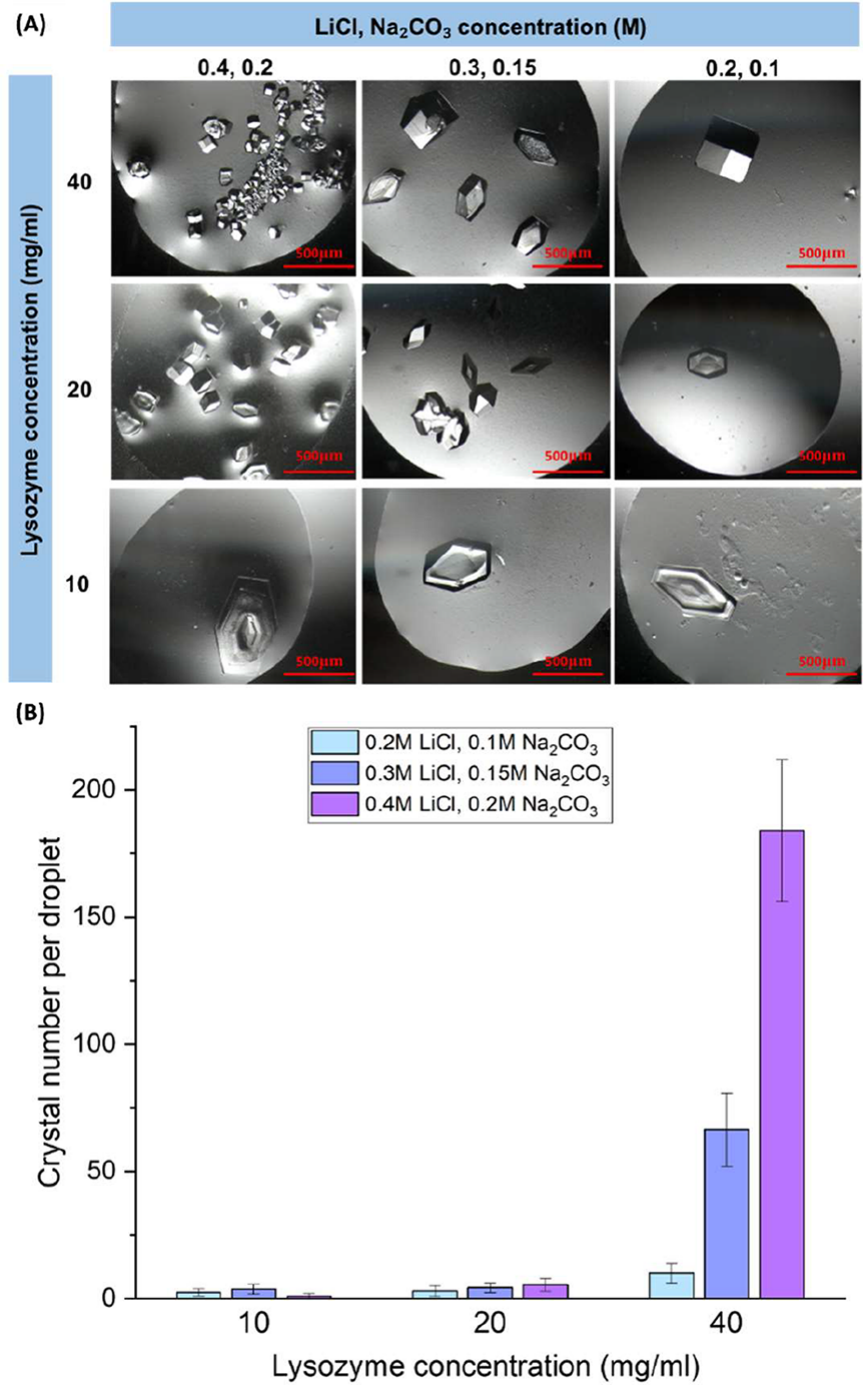
(A) Lysozyme crystals and (B) number of crystals at 20 h in the droplet with lysozyme at concentrations of 10, 20, and 40 mg·mL^–1^, with 0.2, 0.3, and 0.4 M LiCl, and with 0.1, 0.15, and 0.2 M Na_2_CO_3_.

## 4 Discussion

The supersaturation of Li_2_CO_3_ is the strongest influence parameter governing the crystallization process of Li_2_CO_3_ among all factors investigated in this work. For the influence of lysozyme on Li_2_CO_3_ crystallization, it is observed that at the same Li^+^ concentration (e.g., 0.4 M), a decrease in CO_3_^2–^ concentration causes the solution point to shift from the green region (no obvious effects) to the blue region (hindering effects), as shown in Figure 2G. This indicates that with relatively lower CO_3_^2–^, the hindering effect of lysozyme on Li_2_CO_3_ formation is stronger. Under low salt concentrations of LiCl and Na_2_CO_3_ (*S* < 2.4 for Li_2_CO_3_), the proteins in the solution hindered the nucleation. Proteins bind metal cations via electrostatic interactions or surface functional groups.^43–45^ The lysozyme with a positive charge (pI ≈ 11) could bind to CO_3_^2–^. Both the hemoglobin and mRFP, with a negative charge density (pI ≈ 6.8) and (pI ≈ 6.0), respectively, could bind Li^+^. The less Li^+^ or CO_3_^2–^ in the solution resulted in less supersaturation for Li_2_CO_3_ and a slower nucleation process. Hemoglobin has been reported to show strong interactions with metal cations such as Fe^3+^ and Cu^2+^.^46–48^ Modeling studies also indicate that Li^+^ binds to hemoglobin with an affinity more than 12 times that of K^+^,^49^ which is a possible reason that hemoglobin strongly chelates with Li^+^ and hinders the Li^+^ for crystallization, leading to the strongest inhibition effect among the three proteins at low *S*.

Figure 7A illustrates the dual effect of proteins on Li_2_CO_3_ crystallization: with the increase in salt concentrations, proteins become more and more aggregated, which increases the heterogeneous effects and decreases the binding effect, therefore promoting the crystallization of Li_2_CO_3_. At low salt concentrations (*S* < 2.4 for Li_2_CO_3_), proteins are dispersed as small entities in water (e.g., lysozyme dimers). They chelate ions and suppress the nucleation of Li_2_CO_3_, resulting in fewer crystals, as shown in Figure 7B. At moderate salt concentrations (2.4 < *S* < 4.8 for Li_2_CO_3_), proteins aggregate into oligomers. This reduces their ion-binding capacity and weakens their inhibitory effect on nucleation, leading to the formation of more Li_2_CO_3_ crystals, as shown in Figure 7C. At high salt concentrations (*S* > 4.8 for Li_2_CO_3_), the hemoglobin and mRFP protein molecules tended to form large aggregates, similar to the DLS results of lysozyme in Figure 3E,F. The aggregates of protein molecules formed at high *S* further decrease the ability for binding ions, as the specific area is much smaller than that of the dimers and oligomers of protein molecules.^50,51^ On the other hand, the protein aggregates provide more heterogeneous nucleation sites for accelerating the nucleation process of Li_2_CO_3_ (Figure 7D). The mRFP is more stable than lysozyme in neutral and alkaline pH, resulting in fewer aggregates and limited heterogeneous effects. Compared to the lysozyme and mRFP, the hemoglobin is much easier to form tetramers and aggregates, which corresponds to its significant acceleration effect on nucleation time at high *S*.^52^

**Figure 7:**
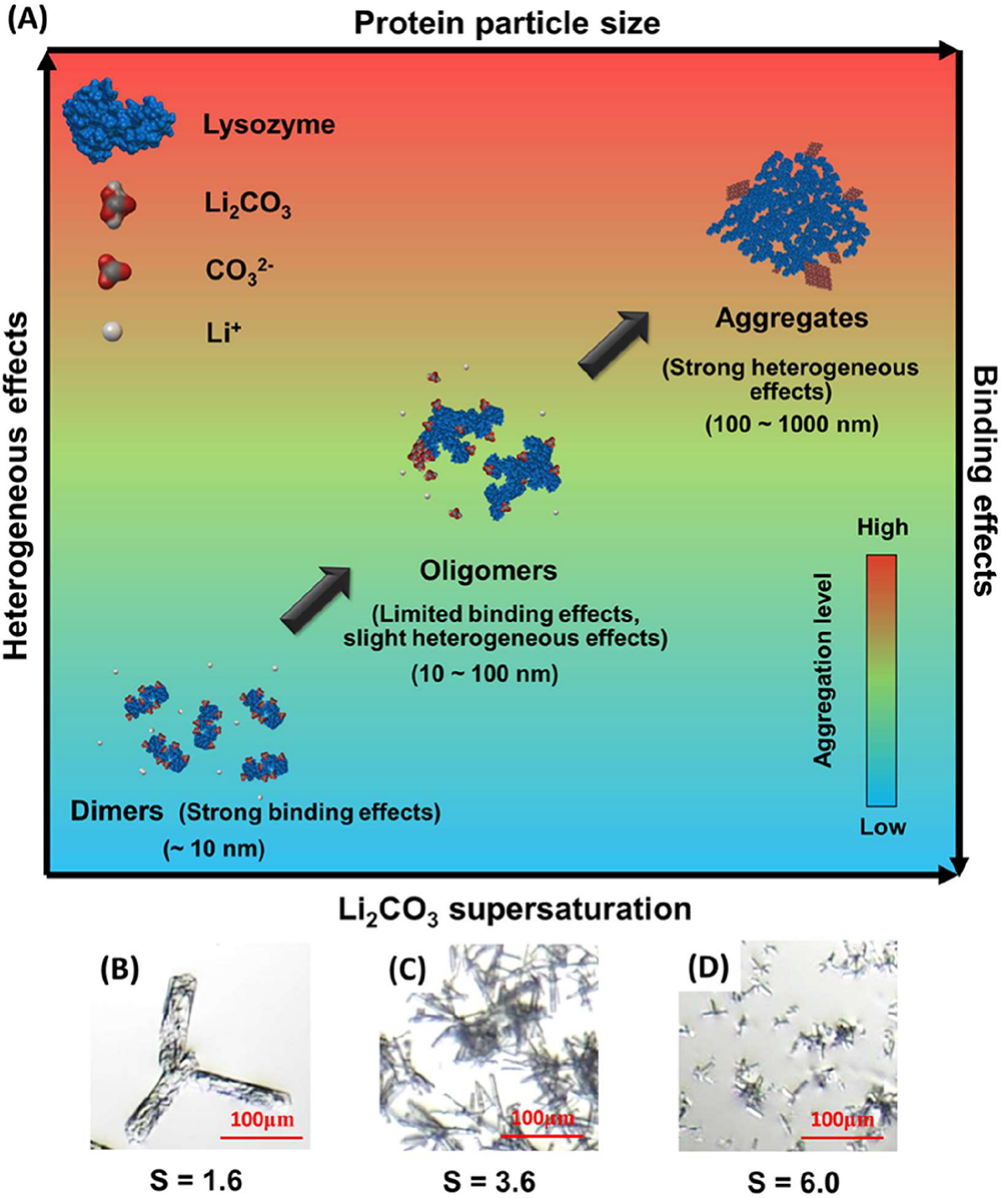
(A) Schematic diagram of protein (i.e., lysozyme) aggregation and enhanced heterogeneous effects with increasing Li_2_CO_3_ concentrations. Li_2_CO_3_ crystals formed under 0.4 M LiCl and 0.3 M Na_2_CO_3_ (B)*, 0.6 M LiCl and 0.3 M Na_2_CO_3_ (C)*, and 0.6 M LiCl and 0.5 M Na_2_CO_3_ (D)*, with 1 mg/mL lysozyme. *Same crystal products shown in Figure 2.

The crystallization processes are dependent not only on the thermodynamic parameters but also on kinetic factors, including solution viscosity and molecular diffusion^53–55^ At low *S*, more protein molecules exist in a dispersed state in the solution. With increasing *S*, protein molecules tend to aggregate, resulting in a reduction in bulk viscosity, as shown in Figure 7. However, the solution is diluted and, therefore, the impact of viscosity on the crystallization kinetics is very limited. On the other side, at lower *S*, the strong interactions between protein molecules and ions hinder the molecular diffusion, hindering the nucleation. At higher *S*, the diffusion rate becomes faster due to the less interactions between protein aggregates and ions.

The SEM image (Figure 8A) shows that Li_2_CO_3_ crystals formed in the droplet without protein have smooth surfaces. Figure 8B shows crystals formed in the droplet with mRFP, which have rough surfaces, suggesting the adsorption of protein on the surface. Similar adsorption phenomena, which result from electrostatic interactions, have been widely reported in lysozyme-CaCO_3_ biomineralization systems and may lead to the incorporation of protein into the precipitate.^56,57^ The Li_2_CO_3_ crystals nucleated with mRFP in Figure 8B exhibited reduced dendritic agglomeration, which is also shown as the influence of lysozyme in Figure 2A–F and the influence of mRFP in Figure 5C. Therefore, protein adsorption could play a crucial role in suppressing the formation of dendritic Li_2_CO_3_ crystals, as proteins on the crystal surface can reduce intercrystalline adhesion resulting from collisions. The phenomenon was consistent with the previous report of lysozyme’s antiagglomeration effects on calcium oxalate.^29^ Lysozyme’s antiagglomeration effect could also be a result of protein adsorption. However, at higher supersaturation, the nucleation occurred very fast (i.e., crystals observed within 2 h at 0.6 M LiCl/0.5 M Na_2_CO_3_ in water), and the effect of lysozyme on Li_2_CO_3_ spherical agglomeration formation was limited. As there were more Li_2_CO_3_ with the same amount of lysozyme, the antiagglomeration effect was relatively weaker, and the fast nucleation led to smaller Li_2_CO_3_ crystals, which were easy to form spherical agglomeration. These findings indicate that the addition of protein can modify the particle size and distribution of Li_2_CO_3_ crystals, thereby improving uniformity and flowability. These attributes can be applied to lithium-related industries, such as manufacturing ceramics, pharmaceuticals, and high-performance lithium batteries.

**Figure 8:**
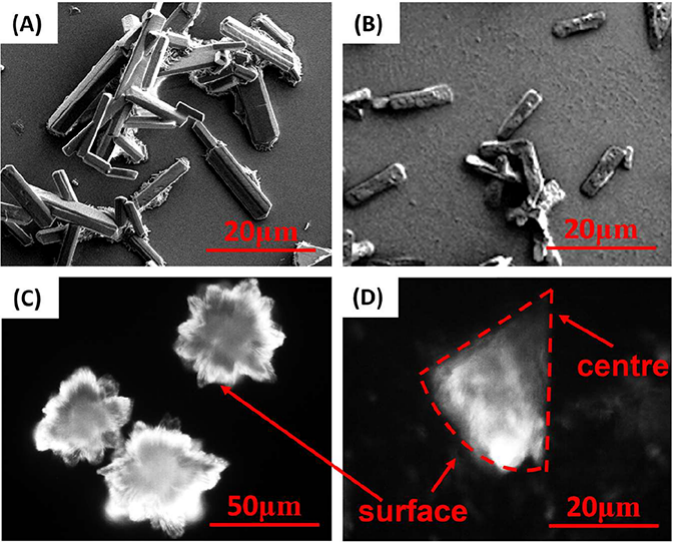
SEM of (A) Li_2_CO_3_ crystals without protein and (B) Li_2_CO_3_ crystals with 25 mg/mL mRFP formed in droplets of 0.6 M LiCl and 0.2 M Na_2_CO_3_. Red fluorescence figures of (C) a spherulite crystal crystallized at 0.6 M LiCl and 0.4 M Na_2_CO_3_ and (D) its cross-sectional view after breaking.

Li_2_CO_3_ forms spherulite crystals in the solution under conditions of 0.6 M LiCl and 0.4 M Na_2_CO_3_, and under the same salt conditions, mRFP could not crystallize or salt out. However, the bright color under fluorescence indicates the protein coexisted with the Li_2_CO_3_ crystals, as shown in Figure 8C. The spherulite crystal was fractured into pieces, and Figure 8D shows a cross-sectional view of the spherulite crystal. The fluorescence intensity of this fan-shaped piece decreases from the surface area to the center area, with no fluorescence signal in the core area. This suggests that proteins could be adsorbed onto the crystal surface after nucleation of Li_2_CO_3_ and form protein–salt composites during the crystal growth, where more protein is observed than in the core. The incorporation of proteins into the salt crystal lattice is difficult due to their large molecular size, whereas the incorporation of salt ions (not salt crystals) into the protein matrix remains possible. Nevertheless, coprecipitation between salt crystals and protein molecules is likely to occur, which was also observed in systems such as CaCO_3_–protein composites and CaP–protein systems.^58,59^

## 5 Conclusions

This work reveals the dual regulatory mechanism of lysozyme, mRFP, and hemoglobin on the nucleation of Li_2_CO_3_. Under low supersaturation of Li_2_CO_3_ (*S* = 2.4), proteins inhibited the nucleation due to ion chelation with the protein dimers, as demonstrated by DLS measurement of lysozyme. At high supersaturation levels of Li_2_CO_3_ (*S* = 4.8), the protein formed precipitates with the disappearance of most dimers and monomers, thereby accelerating nucleation by acting as heterogeneous nucleation sites. Proteins prevent the dendritic and spherical crystal agglomeration of Li_2_CO_3_ through adsorption, with stronger effects by proteins of higher concentrations. However, with a further increase in the concentration of CO_3_^2–^ and Li^+^, the nucleation of Li_2_CO_3_ tended to occur immediately, and the protein showed less antiagglomeration effects within the limited time. The protein formed composites with Li_2_CO_3_ crystals, as proven by the fluorescence signal outside and inside the particles. On the other hand, higher concentrations of CO_3_^2–^ and Li^+^ accelerate the nucleation of proteins, with more lysozyme crystals of smaller size. In addition, the protein can also influence the crystal shape and surface of the Li_2_CO_3_ crystals. These findings provide new insights into protein–salt interactions and offer guidance for the rational control of crystallization processes in biomineralization, advanced material fabrication, and lithium recovery technologies.

## Supplementary Material



## Notes

Distribution of Li_2_CO_3_ nucleation time in neutral pH with/without lysozyme (PDF)

## #

Department of Engineering and Design, School of Engineering and Informatics, University of Sussex, Brighton BN1 9RH, United Kingdom
