# Supplementary material for: Protein Self-Assembly States Modulate Lithium Carbonate Biomineralization: From Ion Chelation to Nucleation Sites
Source: Biomacromolecules. 2025 Dec 12;27(1):428–38. doi: 10.1021/acs.biomac.5c01601 (PMC12801182; doi:10.1021/acs.biomac.5c01601)
Supplement: Supplementary file 1 [file bm5c01601_si_001.pdf]

Supporting Information

Protein Self-Assembly States Modulate Lithium Carbonate Biomineralization: From Ion Chelation to Nucleation Sites

Zhichun Lin[a], Yizhen Yan[b], Archie Hunter[a], Huaiyu Yang[a*]

[a] Department of Chemical Engineering, Loughborough University, Leicestershire, Loughborough, LE11 3TU, United Kingdom

[b] Department of Engineering and Design, School of Engineering and Informatics, University of Sussex, Brighton, BN1 9RH, United Kingdom

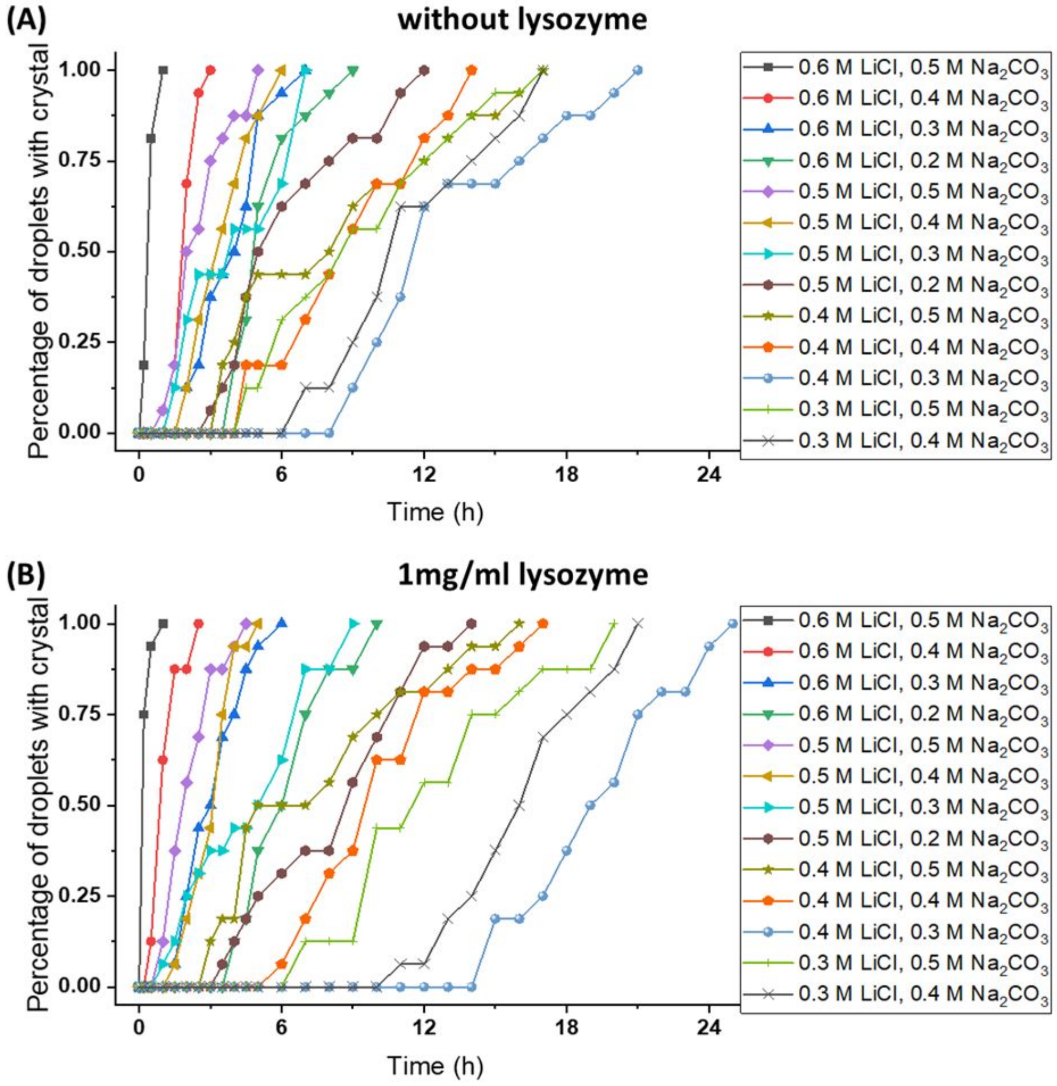

Figure S1 Percentage of droplets with Li_2CO_3 crystals in neutral with 0.3 to 0.6 M LiCl and 0.2 to 0.5 M Na_2CO_3, (A) without lysozyme and (B) with addition of 1 mg/mL lysozyme.
